# 541 A Framework for Multicultural and Multidisciplinary Near-Peer Mentoring for Artificial Intelligence in Healthcare Education: A University of Florida Friend Group – CORRIGENDUM

**DOI:** 10.1017/cts.2024.520

**Published:** 2024-04-23

**Authors:** Daniel Andrew Lichlyter, Myles Joshua T. Tan, Alfredo B. Satriya, Weston J. Schrock, Shaira L. Kee, Michael Aaron G. Sy, Mayra B. Silva, Trevor L. Schrock

The above abstract published with mistakes in some author affiliations.

The correct list of authors and their affiliations is as follows:

Daniel Andrew Lichlyter^1,^*, Myles Joshua Toledo Tan^2,3,4,5,6,^*^,+^, Alfredo Bayu Satriya^2^, Weston John Schrock^4,7^, Shaira Limson Kee^3^, Michael Aaron Go Sy^3^, Mayra Betsabé Silva^8^, Trevor Lamar Schrock^9^

^1^College of Medicine, University of Florida, Gainesville, FL 32610, United States

^2^Department of Electrical & Computer Engineering, Herbert Wertheim College of Engineering, University of Florida, Gainesville, FL 32611, United States

^3^Department of Epidemiology, College of Public Health & Health Professions and College of Medicine, University of Florida, Gainesville, FL 32610, United States

^4^Department of Natural Sciences, College of Arts & Sciences, University of St. La Salle, Bacolod, Negros Occidental 6100, Philippines

^5^Department of Chemical Engineering, College of Engineering & Technology, University of St. La Salle, Bacolod, Negros Occidental 6100, Philippines

^6^Department of Electronics Engineering, College of Engineering & Technology, University of St. La Salle, Bacolod, Negros Occidental 6100, Philippines

^7^College of Pharmacy, University of Florida, Gainesville, FL 32610, United States

^8^Department of Physics, College of Liberal Arts & Sciences, University of Florida, Gainesville, FL 32611, United States

^9^College of Dentistry, University of Florida, Gainesville, FL 32610, United States

*Equal Contribution

^+^Corresponding Author: MylesJoshua.Tan@medicine.ufl.edu

The original abstract has been corrected online to rectify these errors.
